# Pathological phenotypes of astrocytes in Alzheimer’s disease

**DOI:** 10.1038/s12276-023-01148-0

**Published:** 2024-01-04

**Authors:** Junhyung Kim, Ik Dong Yoo, Jaejoon Lim, Jong-Seok Moon

**Affiliations:** 1 Department of Integrated Biomedical Science, Soonchunhyang Institute of Medi-bio Science (SIMS), Soonchunhyang University, Cheonan, 31151 Chungcheongnam-do South Korea; 2 Department of Nuclear Medicine, Soonchunhyang University Hospital Cheonan, Cheonan, 31151 Chungcheongnam-do South Korea; 3 Department of Neurosurgery, Bundang CHA Medical Center, CHA University, Yatap-dong 59, Seongnam, 13496 South Korea

**Keywords:** Cellular neuroscience, Molecular neuroscience

## Abstract

Astrocytes are involved in various processes in the central nervous system (CNS). As the most abundant cell type in the CNS, astrocytes play an essential role in neuronal maintenance and support, synaptic activity, neuronal metabolism, and amyloid-beta (Aβ) clearance. Alzheimer’s disease (AD) is a neurodegenerative disorder associated with cognitive and behavioral impairment. The transformation of astrocytes is involved in various neurodegenerative diseases, such as AD. Since astrocytes have functional diversity and morphological and physiological heterogeneity in the CNS, AD-related astrocytes might show various pathological phenotypes during AD. Astrocytes developing pathological phenotypes could contribute to AD progression. In this review, we provide an overview of the pathological phenotypes of astrocytes in the context of AD, highlighting recent findings in human and mouse AD.

## Unveiling Astrocytes: The Hidden Culprits in Alzheimer’s Disease

Alzheimer’s disease is a widespread type of dementia characterized by memory loss and cognitive dysfunction, with the death of neuronal cells being a key feature. However, the role of astrocytes in the disease’s progression is not fully understood. In a recent study by J.K., J.L., and J.-S.M., they reviewed the pathological phenotypes of astrocytes in Alzheimer’s disease. They explored Alzheimer’s-related astrocytes, discussing the pathological phenotypes in the context of human and mouse Alzheimer’s models. They identified four main phenotypes: reactive, death, senescence, and functional impairment. The study concluded that understanding these pathological phenotypes could help reveal new disease mechanisms of Alzheimer’s and potentially provide alternative treatment approaches. This knowledge could have significant implications for future Alzheimer’s research and treatment strategies. This summary was initially drafted using artificial intelligence, then revised and fact-checked by the author.

## Introduction

Alzheimer’s disease (AD) is a progressive neurodegenerative disease and the most common type of dementia that is characterized by memory loss and cognitive dysfunction^1–3^. The pathological hallmark of AD is the deposition of amyloid-beta (Aβ) plaques and the formation of neurofibrillary tangles (NFTs) that are composed of hyperphosphorylated tau protein^4,5^. Despite numerous studies, the pathophysiological mechanisms of AD are still not fully understood. In contrast, neuronal cell death is a known prominent pathological feature of AD^4,5^. There is a limited understanding of the changes in astrocytes that promote AD pathogenesis.

The diversity of astrocyte populations has been described in different brain regions, and these populations are classified based on morphological and functional features^6–13^. As two large morphological groups, fibrous and protoplasmic astrocytes are located in the white and gray matter of the brain, respectively. Furthermore, astrocytes are classified based on distinct morphological and functional features, such as synapse association^11,12^, membrane properties, Ca^2+^ signaling^12^, and neuronal maturation^13^.

Recent studies have shown that the alteration of astrocytes is involved in the initiation and progression of AD^14,15^. As astrocytes play various physiological roles in synapse formation and function, neurotransmitter release and uptake, the production of trophic factors, and neuronal survival by energetic supports^6,16–19^, the morphological and functional dysregulation of astrocytes is linked to neuronal cell death in AD^20,21^. Understanding AD-related astrocyte subtypes could help identify new pathophysiological mechanisms of AD. In this review, we explore the features of AD-related astrocytes and discuss the pathological phenotypes of astrocytes in the context of humans with AD and mouse AD models.

### The reactive phenotype of astrocytes in AD

#### Proinflammatory, neurotoxic A1 reactive astrocytes

Astrocytes can be changed to reactive astrocytes via morphological, molecular, and functional alterations in various pathological conditions^22,23^. Reactive astrocytes induce neuropathology and neurodegeneration in neurodegenerative diseases and neurotoxic conditions^24^. As part of astrocyte polarization, reactive astrocytes can switch to either the pro-inflammatory, neurotoxic A1 phenotype (A1 astrocytes) or the anti-inflammatory, neuroprotective A2 phenotype (A2 astrocytes)^25,26^.

In a study of reactive astrocytes in the brains of AD patients, both A1 and A2 reactive astrocytes showed upregulated expression of genes such as chitinase 3 like 1 (CHI3L1), ferritin light chain (FTL), integral membrane protein 2 C (ITM2C), aquaporin 4 (AQP4), hepatocellular carcinoma down-regulated 1 (HEPN1), glyceraldehyde-3-phosphate dehydrogenase (GAPDH), angiopoietin-like 4 (ANGPTL4), pleiotrophin (PTN), integral membrane protein 2B (ITM2B), store-operated calcium entry associated regulatory factor (SARAF), interferon-induced transmembrane protein 3 (IFITM3), HtrA serine peptidase 1 (HTRA1), vimentin (VIM), CD63, tweety family member 1 (TTYH1), gap junction protein beta 6 (GJB6), heat shock protein family A (HSP70) member 5 (HSPA5), beta-2 microglobulin (B2M), transmembrane protein 59 (TMEM59), and RAS, dexamethasone-induced 1 (RASD1)^27^. Additionally, reactive astrocytes showed downregulated expression of homeostatic genes such as neurexin 1 (NRXN1), neuregulin 3 (NRG3), glypican 5 (GPC5), and erb-b2 receptor tyrosine kinase 4 (ERBB4)^27^.

As a significant phenotype of reactive astrocytes in AD, A1 reactive astrocytes were identified in the brains of AD patients^28^. A1 reactive astrocytes have high gene levels of glial fibrillary acidic protein (GFAP), S100 calcium-binding protein B (S100B), and complement C3 (C3) in the brains of AD patients^28^.

In studies of reactive astrocytes in mouse AD models, A1 and A2 reactive astrocytes were characterized in the brains of model AD mice^24,29–31^. A1- and A2-reactive astrocytes showed upregulated expression of genes such as lipocalin 2 (Lcn2), six-transmembrane epithelial antigen of prostate 4 (Steap4), sphingosine-1-phosphate receptor 3 (S1pr3), TIMP metallopeptidase inhibitor 1 (Timp1), heat shock protein family B (Small) member 1 (Hspb1), C-X-C motif chemokine ligand 10 (Cxcl10), Cd44, oncostatin m receptor (Osmr), ceruloplasmin (Cp), serine (or cysteine) peptidase inhibitor, clade A, member 3 N (Serpina3n), asparaginase (Aspg), vimentin (Vim), and glial fibrillary acidic protein (Gfap).

A1 reactive astrocytes showed upregulated expression of classical complement cascade genes related to the destruction of synapses^24,30,31^. In the brains of model AD mice, A1 reactive astrocytes had upregulated expression of genes including histocompatibility 2, T region locus 23 (H2-T23), serpin family G member 1 (Serping1), histocompatibility 2, D region locus 1 (H2-D1), glycoprotein alpha-galactosyltransferase 1 (Ggta1), interferon inducible GTPase 1 (Iigp1), guanylate binding protein 2 (Gbp2), fibulin 5 (Fbln5), UDP glucuronosyltransferase family one member A1 (Ugt1a1), FK506 binding protein 5 (Fkbp5), proteasome 20 S subunit beta 8 (Psmb8), serglycin (Srgn), and adhesion molecule with Ig like domain 2 (Amigo2). Additionally, A1 reactive astrocytes produced proinflammatory cytokines such as TNF-α, interleukin (IL)-6, IL-1β, and IL-1α in mouse AD models^31^.

**Fig. 1:**
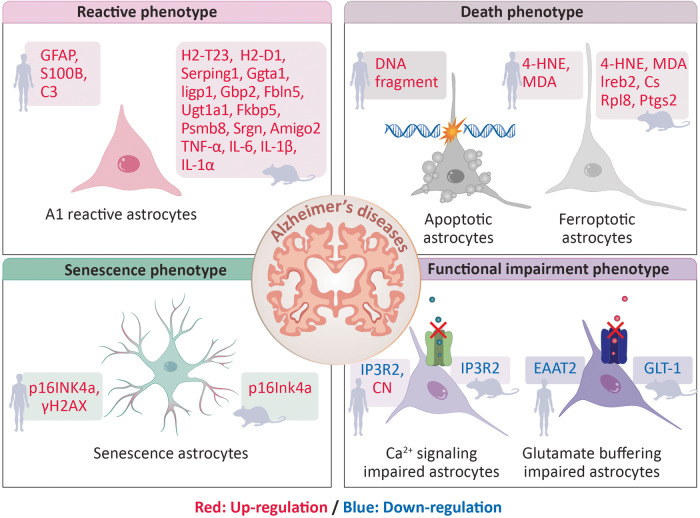
Pathological phenotypes of astrocytes in the context of human and mouse AD. The pathological phenotypes of astrocytes include the reactive phenotype, the death phenotype, the senescence phenotype and functional impairment phenotypes in human and mouse AD. GFAP glial fibrillary acidic protein, S100B S100 calcium binding protein B, C3 complement C3, H2-T23 histocompatibility 2, T region locus 23, H2-D1 histocompatibility 2, D region locus 1, Serping1 serpin family G member 1, Ggta1 glycoprotein alpha-galactosyltransferase 1, Iigp1 interferon inducible GTPase 1, Gbp2 guanylate binding protein 2, Fbln5 fibulin 5, Ugt1a1 UDP glucuronosyltransferase family 1 member A1, Fkbp5 FK506 binding protein 5, Psmb8 proteasome 20 S subunit beta 8, Srgn serglycin, Amigo2 adhesion molecule with Ig like domain 2, TNF-α tumor necrosis factor-alpha, IL-6 interleukin-6, IL-1β interleukin-1 beta, IL-1α interleukin-1 alpha, DNA deoxyribonucleic acid, 4-HNE 4-hydroxynonenal, MDA malondialdehyde, Ireb2, iron response element binding protein 2, Cs citrate synthase, Rpl8 ribosomal protein L8, Ptgs2 prostaglandin-endoperoxide synthase 2, CDKN2A cyclin-dependent kinase inhibitor 2A, γH2AX a phosphorylated form of H2A. X variant histone (H2AX), ITPR2 inositol 1,4,5-trisphosphate receptor type 2, CN calcineurin, EAAT2 glutamate uptake transporter, excitatory amino acid transporters 2, Slc1a2 solute carrier family 1 (glial high-affinity glutamate transporter), member 2.

A2 reactive astrocytes showed upregulated expression of several neurotrophic factors that promote neuronal survival and growth. A2 reactive astrocytes showed upregulated expression of genes including cardiotrophin like cytokine factor 1 (Clcf1), transglutaminase 1 (Tgm1), pentraxin 3 (Ptx3), S100 calcium binding protein a10 (S100a10), sphingosine kinase 1 (Sphk1), Cd109, prostaglandin-endoperoxide synthase 2 (Ptgs2), epithelial membrane protein 1 (Emp1), solute carrier family 10 member 6 (Slc10a6), transmembrane 4 L six family member 1 (Tm4sf1), UDP-GlcNAc:betaGal beta-1,3-*N*-acetylglucosaminyltransferase 5 (B3gnt5), and Cd14^24^. Figure 1 summarizes the phenotypes of A1 reactive astrocytes in human and mouse AD.

### The death phenotypes of astrocytes in AD

#### Apoptotic phenotype of astrocytes

Apoptosis is involved in various diseases of the nervous system^32^. The presence of numerous apoptotic cells is a pathological feature of the brains of patients with AD^33,34^. The levels of the active form of caspase-3 protein, an executioner caspase in apoptosis, were increased in the brains of AD patients^33^. The apoptosis of astrocytes may contribute to the pathogenesis of AD.

In a study on the apoptotic phenotype of astrocytes in the brains of AD patients, the number of DNA fragmentation-positive astrocytes was increased in the temporal lobe of the brain^34^. The number of terminal deoxynucleotidyl transferase dUTP nick end labeling (TUNEL)-positive apoptotic astrocytes was increased in the brains of AD patients^35^. The density of TUNEL-positive astrocytes was correlated with the density of uncored and cored senile plaques, which are polymorphous Aβ protein deposits^35^. TUNEL-positive apoptotic astrocytes showed regressive changes with fragmented processes and cytoplasmic vacuoles in the brains of AD patients^36^. Figure 1 summarizes the apoptotic phenotype of astrocytes in human AD.

#### Ferroptosis phenotype of astrocytes

Ferroptosis is a nonapoptotic form of cell death dependent upon intracellular iron that is morphologically, biochemically, and genetically distinct from other forms of cell death, including apoptosis, necrosis, and autophagy^37^. Ferroptosis is involved in neuronal death during AD pathogenesis^38,39^. Ferroptosis of astrocytes has been identified in AD^38,39^.

In a study on the ferroptosis of astrocytes in AD patients, ferroptosis-related oxidative stress markers, including 4-hydroxynonenal (4-HNE) and malondialdehyde (MDA), were elevated in astrocytes of the cerebral cortex of brains of AD patients^38^. The number of ferroptotic astrocytes was increased in the brains of AD patients^38^. As an upstream molecule involved in ferroptosis in astrocytes, the levels of NADPH oxidase 4 (NOX4) were increased in the 4-HNE-positive astrocytes in the cerebral cortex of brains of AD patients^38^.

In a study of ferroptosis in AD model mice, the levels of the ferroptosis-related proteins 4-HNE and MDA was elevated astrocytes in the cortex of APP/PS1 AD model mice^38^. Furthermore, the number of ferroptotic astrocytes was increased in the cortex of APP/PS1 AD model mice^38^. The expression of ferroptosis-related genes, such as iron response element binding protein 2 (Ireb2), citrate synthase (Cs), ribosomal protein L8 (Rpl8), and prostaglandin-endoperoxide synthase 2 (Ptgs2), was upregulated in hippocampal tissues of APP/PS1 AD model mice^39^. Figure 1 summarizes the phenotypes of ferroptotic astrocytes in human and mouse AD.

### The senescence phenotype of astrocytes in AD

Aging is a primary risk factor in the pathogenesis of AD^40^. Cellular senescence is the hallmark of aging^41,42^. Cellular senescence in the brain may link the aging process to AD progression. The senescence of astrocytes is related to AD pathogenesis^43^.

In a study on the senescence of astrocytes in AD patients, senescent astrocytes showed upregulated expression of cyclin-dependent kinase inhibitor 2 A (CDKN2A) (also known as p16INK4a), which is a marker of senescence, in the frontal cortex of AD patients^44^. The number of senescent astrocytes was increased in AD patients^44^. As a marker of aging-related DNA damage, the expression of γH2AX, a phosphorylated form of H2A. X variant histone (H2AX), which is a part of the nucleosome structure, was increased in the hippocampal regions and cerebral cortex of AD patients^45^. γH2AX is produced through phosphorylation in response to the formation of double-stranded breaks in chromosomal DNA^46–48^. Furthermore, the phosphorylation of H2AX induces the translocation of phosphatidylserine to the outer cell membrane and the internucleosomal DNA fragments during apoptosis^49^.

In a study on the senescence of astrocytes in mouse AD, the expression of senescence-associated genes such as Cdkn2a was increased in the astrocytes of tau MAPT P301S PS19 transgenic mice^44^. Pharmacological elimination of senescent astrocytes by the senolytic agent ABT263 prevented the upregulation of senescence-associated gene expression and attenuated tau phosphorylation in the brains of tau MAPT P301S PS19 transgenic mice^50–52^. Figure 1 summarizes the phenotypes of senescent astrocytes in human and mouse AD.

### The functional impairment of astrocytes in AD

#### Impaired Ca^2+^ signaling function in astrocytes during AD

Astrocytes play a role in maintaining the homeostasis of neuronal circuits and regulating neuronal activity via intracellular calcium signals^6,53,54^. Dysfunction of astrocyte calcium signaling leads to network hyperactivity in the early stage of AD^55^. Dysregulation of calcium signaling in astrocytes might contribute to the pathological progression of AD.

In the study of astrocyte calcium signaling in AD patients, the expression of inositol 1,4,5-trisphosphate receptor type 2 (ITPR2) (also known as IP3 receptor type 2 (IP3R2)), an intracellular calcium release channel, was decreased in the astrocytes in brains of AD patients^55,56^. Furthermore, the levels and nuclear localization of nuclear factor of activated T cells 3 (NFAT3), which regulates calcineurin (CN), a Ca^2+^/calmodulin-dependent protein phosphatase, were elevated in the brains of AD patients^57^. Elevation of CN in astrocytes triggered the induction of genes associated with inflammatory responses in early-stage AD patients^58^.

In a study on calcium signaling in astrocytes in mouse AD, decreased calcium signaling was caused by a reduction in Itpr2 expression in astrocytes before the accumulation of Aβ plaques in the early stage of AD in App^NL-F^ model mice^55,56^. Figure 1 summarizes the phenotypes of Ca^2+^ signaling-impaired astrocytes in human and mouse AD.

#### Impaired glutamate buffering in astrocytes during AD

Glutamate is an excitatory neurotransmitter in the brain. Glutamate receptor activation by interaction with glutamate contributes to various neurologic functions, such as cognition, memory, movement, and sensation^59,60^. Astrocytes play a significant role in glutamate uptake by clearing 80 ~ 90% of synaptic glutamate in the synaptic cleft;^61–65^ glutamate uptake transporters, excitatory amino acid transporters 1 (EAAT1) and 2 (EAAT2) (known in mouse as Slc1a3 solute carrier family 1 (glial high-affinity glutamate transporter), member 3 (Slc1a3) (also known as GLAST) and solute carrier family 1 (glial high-affinity glutamate transporter), member 2 (Slc1a2) (also known as GLT-1), respectively) were expressed in astrocytes of AD patients^66–69^. EAAT-1 and EAAT-2 are localized in perisynaptic astrocytes and are in contact with active synapses of glutamatergic neurons^70^. Slc1a3 and Slc1a2 are localized in astrocytic soma^68,69^. Glutamate excitotoxicity has implications for neurodegeneration in AD^71^.

In a study on glutamate uptake in astrocytes in AD patients, glutamate transport activity was reduced in the cortex of brains of AD patients^72^. The expression of the EAAT2 protein and glutamate transport activity were decreased in the frontal cortex of AD patients^73^. Additionally, a reduction in glutamate transport activity was associated with enhanced Aβ accumulation in AD patients^73^.

In a study on glutamate uptake in the astrocytes of AD mice, the loss of Slc1a2 exacerbated cognitive impairment in the AD model mice^74^. Additionally, restoration of Slc1a2 function improved cognitive functions, restored synaptic integrity and reduced amyloid plaques in a AD model mice^75^. Figure 1 summarizes the phenotypes of impaired glutamate buffering in astrocytes in human and mouse AD.

## Conclusions

We reviewed the pathological phenotypes of astrocytes in AD and discussed the transcriptomic and proteomic features of the pathological phenotypes of astrocytes in the brains of AD patients and AD model mice. We described the reactive phenotype, death phenotype, senescence phenotype, and functional impairment phenotypes of astrocytes in human and mouse AD. The development of pathological phenotypes by astrocytes may be an essential event in the pathogenesis of AD. Understanding the pathological phenotypes of astrocytes may help maintain normal brain function and prevent neurodegeneration during AD. Along with current therapies for AD that target Aβ and tau pathology, the proper control of astrocyte pathology could be an alternative therapeutic approach for AD treatment.
